# *Sedum hengshanense* (Crassulaceae), a new species from Hunan, central China

**DOI:** 10.3897/phytokeys.279.192928

**Published:** 2026-09-23

**Authors:** Xiang-Dong Qiu, Jiang-Lin Xia, Feng-Di Zhang, Guo-Ping Wang, Guo-Ping Tan, Yu Xiong, Sheng-Wei Wang, Neng Wei, Qing-Feng Wang

**Affiliations:** 1 State Key Laboratory of Plant Diversity and Specialty Crops, Wuhan Botanical Garden, Chinese Academy of Sciences, Wuhan, Hubei 430074, China State Key Laboratory of Plant Diversity and Specialty Crops, Wuhan Botanical Garden, Chinese Academy of Sciences Wuhan China; 2 School of Ecology and Environment, Tibet University, Lhasa 850032, Xizang, China School of Ecology and Environment, Tibet University Lhasa China; 3 University of Chinese Academy of Sciences, Beijing 100049, China University of Chinese Academy of Sciences Beijing China; 4 Nanyue Arboretum of Hunan Province, Nanyue 421900, Hunan, China Nanyue Arboretum of Hunan Province Nanyue China; 5 Matoushan National Nature Reserve, Zixi 335300, Jiangxi, China Matoushan National Nature Reserve Zixi China; 6 Sino-Africa Joint Research Center, Chinese Academy of Sciences, Wuhan, Hubei 430074, China Sino-Africa Joint Research Center, Chinese Academy of Sciences Wuhan China

**Keywords:** Hengshan Mountain, phylogeny, succulent plant, taxonomy

## Abstract

*Sedum hengshanense*, a new species of Crassulaceae from central China, is described and illustrated. Morphological evidence supports its placement in *Sedum* sect. *Sedum*. The new species is morphologically similar to *S. spiralifolium*, *S. sarmentosum*, *S. lineare*, *S. polytrichoides* and *S. morrisonense*, but differs by its alternate phyllotaxy, leaf apex not twisted when young, papillate leaf margin, longer basal leaf spurs (1.0–1.4 mm long) with an acuminate apex, petals with the apex mucronate at the tip and broadly spatulate to subtrapezoid, obtuse-apexed nectar scales. Phylogenetic analysis recovers *S. hengshanense* as sister to the clade comprising *S. spiralifolium*, *S. sarmentosum* and *S. lineare*. The species is assessed as Vulnerable (VU) according to the IUCN Red List Categories and Criteria.

## Introduction

*Sedum* L., a member of the subfamily Sempervivoideae, is the largest genus in Crassulaceae, comprising approximately 490 accepted species (POWO 2026). It is widely distributed across all continents, except Antarctica, with major centres of diversity in the Euro–Mediterranean Region, West–Central Asia, East Asia and North America (particularly the USA and Mexico) (Thiede and Eggli 2007; POWO 2026). Species of *Sedum* are succulent herbs typically adapted to arid and/or rocky habitats (Thiede and Eggli 2007). Some species have ornamental value, such as *S. acre* and *S. lineare*, whereas others are used in traditional medicine, including *S. sarmentosum* and *S. praealtum* (Ma et al. 2017; Boriollo et al. 2020). In addition, several species, such as *S. plumbizincicola*, have a strong capacity for heavy metal accumulation and show potential for applications in soil remediation (Wang et al. 2023).

China harbours a rich diversity of *Sedum*. Recent studies indicate that around 148 species occur in China, including 118 endemics (Dai et al. 2025; Huang et al. 2025; Yi et al. 2025; She et al. 2026). Of these, 27 species have been described as new to science over the past two decades. These recent discoveries indicate that the diversity of *Sedum* species in China remains underestimated and that further taxonomic exploration is needed. In April 2024, we conducted a field survey in Hengshan Mountain, Hunan Province, China, where a previously unknown perennial caespitose *Sedum* species was discovered in flower. This species was clearly distinct from other *Sedum* species encountered at the site, including *S. grammophyllum*, *S. sarmentosum*, *S. emarginatum*, *S. bulbiferum* and *S. alfredii*. Over the following two years, successive field investigations were carried out to further document its distribution and morphological variation. Specimens were collected and living plants were transplanted to Wuhan Botanical Garden and Nanyue Arboretum of Hunan Province for detailed morphological examination. Based on comparative morphological studies and molecular phylogenetic analyses, the species is formally described as a new species, *Sedum hengshanense*.

## Materials and methods

### Morphological study

We conducted field surveys over two years in Hengshan Mountain, Hunan Province, to investigate the morphological variation, geographic distribution and phenology of the species in its natural habitat. Based on these observations, we collected voucher specimens and samples for molecular analyses during the flowering and fruiting periods. Additionally, detailed anatomical observations and video recordings of the flowers were made. Living plants were also collected and cultivated at Nanyue Arboretum of Hunan Province and Wuhan Botanical Garden, Chinese Academy of Sciences, where the plants are currently thriving. Morphological characteristics were examined and photographed using a Zoom Stereo Microscope-TS-10W (PDV, China) and an EOS M5 Canon camera. The morphology of pollen and seed was analysed and photographed with a HITACHI TM3030 scanning electron microscope (SEM) (Hitachi, Ltd., Japan).

To evaluate whether the new species had previously been collected or overlooked, we examined *Sedum* specimens and specimen records available through the Chinese Virtual Herbarium (CVH), including specimens mainly deposited in PE, IBSC, KUN, NAS, WUK, LBG, SCFI and other herbaria in China. We also examined our own field collections deposited in HIB and PE. Morphological comparisons were made, based on field observations, cultivated plants, herbarium specimens and relevant taxonomic literature.

### Molecular study

The nuclear ribosomal internal transcribed spacer region (ITS) was used for the phylogenetic analyses because it has been widely employed in phylogenetic studies of *Sedum* (Lu and Wang 2023; Chai et al. 2024; Yi et al. 2025; She et al. 2026). A total of 81 ITS sequences representing accessions within Crassulaceae were used to reconstruct phylogenetic trees and infer the phylogenetic position of the new species. Amongst these, two sequences of this newly-discovered species, obtained from samples collected from two populations on Hengshan Mountain, were newly generated in this study, whereas the remaining 79 sequences were downloaded from the National Center for Biotechnology Information (NCBI; https://www.ncbi.nlm.nih.gov/). Of the 81 sequences included in the analysis, 79 represent taxa of *Sedum*, while *Aeonium viscatum* and *Greenovia aizoon* were selected as outgroups following Yi et al. (2025). Details of the materials, including their sources and GenBank accession numbers, are presented in Table 1.

**Table 1.** Taxa included in this study with voucher information, GenBank accession numbers and references. Missing data are indicated with “–”.

**Taxon**	**Vouche**r	**Accession number**	**Reference**
*S. actinocarpum*	Ito 1749	LC229265	Ito et al. (2017a)
*S. alfredii*	IBK194564	FJ919949	Wang and Shu unpublished
*S. alfredii*	IBK114924	FJ919951	Wang and Shu unpublished
*S. arisanense*	Ito 1842	LC229273	Ito et al. (2017a)
*S. arisanense*	Ito 1836	LC229272	Ito et al. (2017a)
*S. baileyi*	LBG0064555	FJ919935	Wang and Shu unpublished
*S. bergeri*	–	AY352897	Ni et al. unpublished
*S. boninense*	Ito 2371	LC229242	Ito et al. (2017a)
*S. bulbiferum*	Ito 416	LC229234	Ito et al. (2017a)
*S. bulbiferum*	130514hs41	KM111166	Xie et al. (2014)
*S. bulbiferum*	130524qz09	KM111165	Xie et al. (2014)
*S. brachyrinchum* var. brachyrinchum	Ito 1359	LC229274	Ito et al. (2017a)
*S. danjoense*	Ito 3658	LC260127	Ito et al. (2017b)
*S. emarginatum*	130512hs27	KM111145	Xie et al. (2014)
*S. emarginatum*	130529hz03	KM111146	Xie et al. (2014)
*S. emarginatum*	130503jz21	KM111147	Xie et al. (2014)
*S. emarginatum*	24041001	PP981038	Dai et al. (2025)
*S. emarginatum*	24052201	PP981037	Dai et al. (2025)
*S. emarginatum*	AYJT-Nanjing-0308-08	EU592006	–
*S. erici-magnusii*	Ito 2077	LC229235	Ito et al. (2017a)
*S. erythrospermum*	Tsutsumi 1504	AB906473	Ito et al. (2014b)
*S. formosanum*	Ito1115	LC530813	Ito et al. (2020)
*S. formosanum*	Ito2296	LC260124	Ito et al. (2017b)
*S. hakonense*	Mayuzumi C00005	AB088625	Mayuzumi and Ohba (2004)
*S. hangzhouense*	Ito 2604	LC260130	Ito et al. (2017b)
*S. hengshanense* 1	QS16	PZ161008	**This study**
*S. hengshanense* 2	QS112	PZ161009	**This study**
*S. japonicum*	Kokubugata 16749	AB906475	Ito et al. (2014b)
*S. japonicum* var. *oryzifolium*	Ito 2285	LC229239	Ito et al. (2017a)
*S. japonicum* var. *pumilum*	Ito 2287	LC229240	Ito et al. (2017a)
*S. japonicum* var. *senanense*	Ito 2200	LC229238	Ito et al. (2017a)
*S. jinglanii*	Y. S. Huang 21040301	OP288035	Huang et al. (2023)
*S. jinglanii*	DNPC 2873	OQ162326	Huang et al. (2023)
*S. jiulungshanense*	Ito 76	LC229243	Ito et al. (2017a)
*S. kiangnanense*	CMQ1030	LC229244	Ito et al. (2017a)
*S. lineare*	Mayuzumi C00120	AB088623	Mayuzumi and Ohba (2004)
*S. lineare*	TNS: Ito667	LC229245	Ito et al. (2017b)
*S. lipingense*	ZRB1479	MN150061	Zhang et al. (2019)
*S. lungtsuanense*	Ito 3563	LC260131	Ito et al. (2017b)
*S. makinoi*	Kokubugata 16730	AB906476	Ito et al. (2014b)
*S. mexicanum*	Ito 647	LC229247	Ito et al. (2017a)
*S. microsepalum*	Ito 2771	LC229282	Ito et al. (2017a)
*S. microsepalum*	Ito 1965	LC229281	Ito et al. (2017a)
*S. morrisonense*	Ito 2765	LC229290	Ito et al. (2017a)
*S. morrisonense*	Hornat S1	LM993281	Nikulin et al. (2016)
*S. multicaule*	Miyamoto et al. TI9596136	AB088631	Mayuzumi and Ohba (2004)
*S. nagasakianum*	Ito 2064	LC229249	Ito et al. (2017a)
*S. nokoense*	Kokubugata 10426	AB906478	Ito et al. (2014b)
*S. oligospermum*	Ito 74	LC229250	Ito et al. (2017a)
*S. oreades*	Rao 090803-03	KF113733	Zhang et al. (2014)
*S. spiralifolium*	130425sc01	KM111158	Xie et al. (2014)
*S. spiralifolium*	130425sc02	KM111159	Xie et al. (2014)
*S. spiralifolium*	130423zy62	KM111163	Xie et al. (2014)
*S. polytrichoides* var. polytrichoides	CMQ1057	LC229251	Ito et al. (2017a)
*S. polytrichoides* var. *setouchiense*	Ito 2298	LC229253	Ito et al. (2017a)
*S. rupifragum*	Ito2070	LC229254	Ito et al. (2017a)
*S. sarmentosum*	130529lq03	KM111155	Xie et al. (2014)
*S. sarmentosum*	130511hs22	KM111156	Xie et al. (2014)
*S. sarmentosum*	130531la22	KM111157	Xie et al. (2014)
*S. satumense*	Ito 2295	LC229256	Ito et al. (2017a)
*S. sekiteiense*	Ito 1456	LC229295	Ito et al. (2017a)
*S. simingshanense*	–	PP464049	She et al. (2025)
*S. simingshanense*	–	PP464048	She et al. (2025)
*S. subtile*	Ito 624	AB930277	Ito et al. (2014a)
*S. subtile*	Shimizu 1999	AB088622	Mayuzumi and Ohba (2004)
*S. taiwanianum*	Ito 2770	LC229297	Ito et al. (2017a)
*S. taiwanianum*	Ito 2770	LC229296	Ito et al. (2017a)
*S. tetractinum*	Ito 3623	LC260135	Ito et al. (2017b)
*S. tianmushanense*	Ito 355	LC229261	Ito et al. (2017a)
*S. tosaense*	Kokubugata 16726	AB906483	Ito et al. (2014b)
*S. triactina*	9596091	AB088629	Mayuzumi and Ohba (2004)
*S. tricarpum*	Ito 2269	LC229259	Ito et al. (2017a)
*S. tricarpum*	–	PP989617	–
*S. trullipetalum*	Miyamoto et al. 9420132	AB088630	Mayuzumi and Ohba (2004)
*S. truncatistigmum*	Ito 3254	LC229306	Ito et al. (2017a)
*S. xunvense*	–	PV101948	Chai et al. (2024)
*S. xunvense*	–	PV101947	Chai et al. (2024)
*S. yabeanum*	Ito 396	AB906490	Ito et al. (2014b)
*S. zentaro-tashiroi*	Ohba 1998	AB088619	Mayuzumi and Ohba (2004)
*Aeonium viscatum*	Mort 1432	AY082154	Mort et al. (2002)
*Greenovia aizoon*	Mort 1425	AY082112	Mort et al. (2002)

Fresh leaves from two individuals of *S. hengshanense* were dried in silica gel and total genomic DNA was extracted using a modified CTAB method (Doyle and Doyle 1987). DNA quality was checked by 1% agarose gel electrophoresis. The ITS region was amplified with ITS1 and ITS4 primers (White et al. 1990), which have been commonly used in phylogenetic studies of *Sedum* (Huang et al. 2023; Ito et al. 2023; Yi et al. 2025). PCR amplification was performed following Ito et al. (2023) with minor modifications: 94°C for 3 min; 35 cycles of 94°C for 30 s, 50°C for 30 s and 72°C for 90 s; and a final extension at 72°C for 7 min. PCR products were checked on 1% agarose gels, purified and sequenced in both directions using the same primers. Forward and reverse reads were assembled and checked manually in Geneious Prime v.2025.0.3.

All ITS sequences were aligned using MAFFT v.7 (Katoh and Standley 2013) with default parameters and poorly-aligned regions were removed with trimAl (Capella-Gutiérrez et al. 2009). Alignments were then visually inspected and manually adjusted, when necessary, in Geneious Prime v.2025.0.3 (Kearse et al. 2012). Maximum-Likelihood (ML) analysis was conducted using IQ-TREE v.2.2.0. The best-fitting nucleotide substitution model was selected using ModelFinder according to the Bayesian information criterion (BIC; Kalyaanamoorthy et al. 2017) and SYM+I+G4 was selected as the optimal model. Branch support was assessed using 5,020 ultrafast bootstrap replicates. Bayesian-Inference (BI) analysis was conducted using MrBayes v.3.2.6 (Ronquist et al. 2012) under the GTR+I+G model. Two independent runs, each with four Markov chains, were conducted for 2,000,000 generations, with trees sampled every 1,000 generations. The first 25% of sampled trees were discarded as burn-in. Convergence was assessed by confirming that the average standard deviation of split frequencies was below 0.01 and that effective sample sizes (ESS) for all parameters exceeded 200. The resulting phylogenetic trees were visualised and annotated using the Interactive Tree of Life (iTOL; Letunic and Bork 2007).

## Results

### Morphological comparisons

The carpels and follicles of *Sedum hengshanense* are adaxially gibbous. According to the sectional circumscription of Fu and Ohba (2001), the species is, therefore, placed in *Sedum* sect. *Sedum*. The species is readily recognised by its alternate and imbricate leaves, a long basal leaf spur, leaf margins with conspicuous papillae and broadly spatulate to subtrapezoid nectar scales with obtuse apices.

Morphologically, *S. hengshanense* is similar to *S. spiralifolium*, *S. sarmentosum*, *S. lineare*, *S. polytrichoides* and *S. morrisonense*. *Sedum hengshanense* shares with *S. spiralifolium*, *S. sarmentosum* and *S. lineare* a perennial habit, the presence of a basal leaf spur, petals and nectar scales of comparable size and papillate seed surfaces. However, the new species differs from these species in a combination of leaf arrangement, leaf texture, leaf shape and size, the length and apex shape of the basal leaf spur, the presence of conspicuous papillae along the leaf margins and papillate sepal apices (Table 2). *Sedum hengshanense* also resembles *S. polytrichoides* and *S. morrisonense* in having erect flowering stems, alternate leaves and young leaves not twisted at the apex. However, it differs from both species in having papillate leaf margins, papillate sepal apices and petals with the apex mucronate at the tip. In addition, *S. hengshanense* differs from *S. polytrichoides* in having oblong-lanceolate rather than linear to linear-lanceolate leaves and longer basal leaf spurs (1.0–1.4 mm vs. ca. 0.8 mm). It differs from *S. morrisonense* in having oblong-lanceolate, thinner and narrower leaves rather than oblanceolate to linear-spatulate, thicker and broader leaves and longer basal leaf spurs with acuminate rather than obtuse apices (Table 2).

**Table 2.** Comparison of *S. hengshanense* and its morphologically similar species. Missing data are indicated with “–”.

**Character**	***S. hengshanense***	***S. spiralifolium***	***S. sarmentosum***	***S. lineare***	***S. polytrichoides***	***S. morrisonense***
**Flowering stem**	Erect	Erect	Procumbent	Erect	Erect	Erect
**Phyllotaxy**	Alternate, imbricate	3-verticillate	3-verticillate	3(4)-verticillate	Alternate	Alternate, imbricate
**Tender leaf**	Not twisted at apex	Apex twisted	Not twisted at apex	Not twisted at apex	Not twisted at apex	Not twisted at apex
**Leaf blade**	Oblong-lanceolate	Linear-lanceolate	Oblanceolate to oblong	Linear	Linear to linear-lanceolate	Oblanceolate to linear-spatulate
**Leaf margin**	Papillate	Not papillate	Not papillate	Not papillate	Not papillate	Not papillate
**Basal leaf spur**	1.0–1.4 mm long, apex acuminate	1.0–1.6 mm long, mostly 3-lobed, middle tilted	Ca. 1 mm long, apex obtuse	Ca. 0.9–1.2 mm long, apex subacute	Ca. 0.8 mm long, apex obtuse to truncate	Ca. 0.5 mm long, apex obtuse
**Leaf (length × width)**	7–10 × 1.5–2.0 mm	10–25 × 2–3 mm	15–28 × 3–7 mm	10–15 × ca. 2 mm	5–11 × 1–2 mm	10–15 × 2.5–3 mm
**Leaf thickness**	Ca. 0.6 mm	Ca. 1 mm	Ca. 1.5 mm	Ca. 0.7 mm	Ca. 0.7 mm	Ca. 1 mm
**Sepal**	Papillate at apex	Not papillate at apex	Not papillate at apex	Not papillate at apex	Not papillate at apex	Not papillate at apex
**Petal**	5–6 × 1.2–1.5 mm, apex mucronate at the tip	4–7 × 1–2 mm, apex mucronate at the tip	5–8 × 1–2 mm, apex mucronate at the tip	4–6 × 1.0–1.5 mm, apex not mucronate at the tip	5–7 mm, apex not mucronate at the tip	4–6 × ca. 1.2 mm, apex not mucronate at the tip
**Nectar scale**	Broadly spatulate to subtrapezoid, apex obtuse	Nearly fan-shaped, apex obtuse	Cuneate-quadrangular, apex subemarginate	Broadly cuneate to subquadrangular, apex obtuse	Broadly rounded-cuneate, base cuneate, apex broadly rounded	Obovate
**Nectar scale size**	Ca. 0.5 × 0.3 mm	Ca. 0.5 × 0.2 mm	Ca. 0.5 × 0.5 mm	Ca. 0.5 × 0.5–0.6 mm	Ca. 0.3 × 0.3 mm	–
**Flowering period**	Apr–May	Apr	May–Jul	Apr–May	Jul–Aug	Jul–Aug

### Molecular phylogenetic analyses

**Figure 1.**
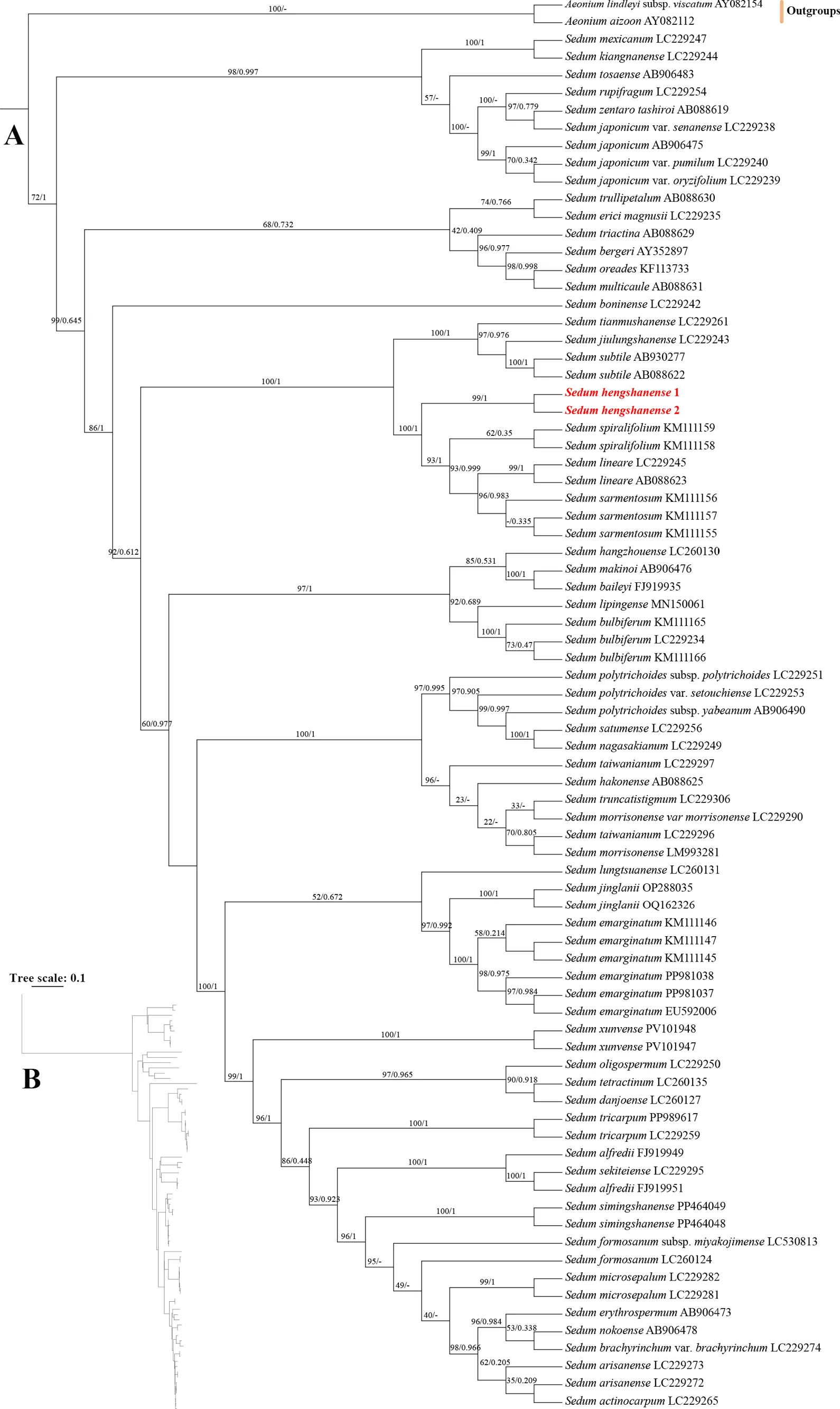
**A**. Maximum Likelihood phylogeny of *Sedum hengshanense* and its relatives inferred from ITS sequences, with the new species shown in bold red; **B**. The same tree as **A** showing branch lengths proportional to nucleotide substitutions per site.

Based on the ITS dataset, we reconstructed both Maximum-Likelihood (ML) and Bayesian-Inference (BI) phylogenetic trees. The two analyses yielded largely congruent topologies and the branch support values (bootstrap support, BS) and posterior probabilities (PP) for each node are shown on the ML tree (Fig. 1). *Sedum hengshanense* forms a distinct lineage and is sister to the clade comprising *S. spiralifolium*, *S. sarmentosum* and *S. lineare*, with full support (BS = 100%, PP = 1). This molecular evidence further supports *S. hengshanense* as a distinct species.

## Discussion

The recognition of *Sedum hengshanense* as a distinct species is supported by both morphological and molecular phylogenetic evidence. Although the species is morphologically similar to several related species, including *S. spiralifolium*, *S. sarmentosum*, *S. lineare*, *S. polytrichoides* and *S. morrisonense*, it can be consistently distinguished by a combination of diagnostic morphological characters (Table 2). These morphological differences were consistently observed across the examined populations and specimens. Furthermore, the ITS phylogeny recovered *S. hengshanense* as a distinct lineage that is sister to the clade comprising *S. spiralifolium*, *S. sarmentosum* and *S. lineare*, with strong support (Fig. 1). Together, these results provide complementary evidence for the recognition of *S. hengshanense* as a distinct species.

Morphologically, *S. hengshanense* is assignable to *Sedum* sect. *Sedum* sensu Fu and Ohba (2001), a section comprising more than 60 species, of which 49 are recorded from China. In recent years, approximately 20 new species assignable to this section have been described from East Asia, particularly China (Dai et al. 2025; Yi et al. 2025), indicating that the species diversity of this section remains incompletely documented. The discovery of *S. hengshanense*, therefore, further highlights the high taxonomic diversity of *Sedum* in East Asia and the need for continued field and taxonomic investigations in the region.

The ITS region was used in this study because sequence data are widely available for *Sedum* and the marker has been widely employed in phylogenetic studies of the genus. Its use also enabled relatively broad taxon sampling, based on sequences available in public databases. However, the use of a single molecular marker and limited intraspecific sampling, comprising one ITS sequence from each of two sampled populations, restricts our ability to assess population-level genetic variation and fine-scale phylogenetic relationships. Although the current ITS data support the inferred phylogenetic placement of *S. hengshanense*, broader population-level sampling encompassing all known populations, together with additional molecular markers, will be necessary to more comprehensively assess its infraspecific genetic diversity and phylogenetic coherence across its known range.

### Taxonomic treatment

#### Sedum hengshanense

Taxon classificationPlantaeSaxifragalesCrassulaceae

N.Wei, X.D.Qiu, J.L.Xia & Q.F.Wang sp. nov.

991343C9-9635-56D8-85BC-08752E956CC5

urn:lsid:ipni.org:names:77397020-1

Figs 2, 3

##### Chinese name.

衡山景天.

##### Type.

China • Hunan: Nanyue District, Hengshan Mountain, Zhurong Peak, alt. 1245 m, 27.293782, 112.694611, on a rocky slope, 28 Apr 2025, *J.L.Xia QS112* (holotype: HIB!; isotype: PE!).

##### Diagnosis.

*Sedum hengshanense* is morphologically most similar to *S. morrisonense*, but differs in having papillate leaf margins (vs. non-papillate leaf margins), smaller and thinner leaves (7–10 × 1.5–2.0 mm, ca. 0.6 mm thick vs. leaves 10–15 × 2.5–3 mm, ca. 1 mm thick), longer basal leaf spurs (1.0–1.4 mm long vs. ca. 0.5 mm long) with an acuminate apex (vs. obtuse apex) and broadly spatulate to subtrapezoid nectar scales with an obtuse apex (vs. obovate nectar scales).

**Figure 2.**
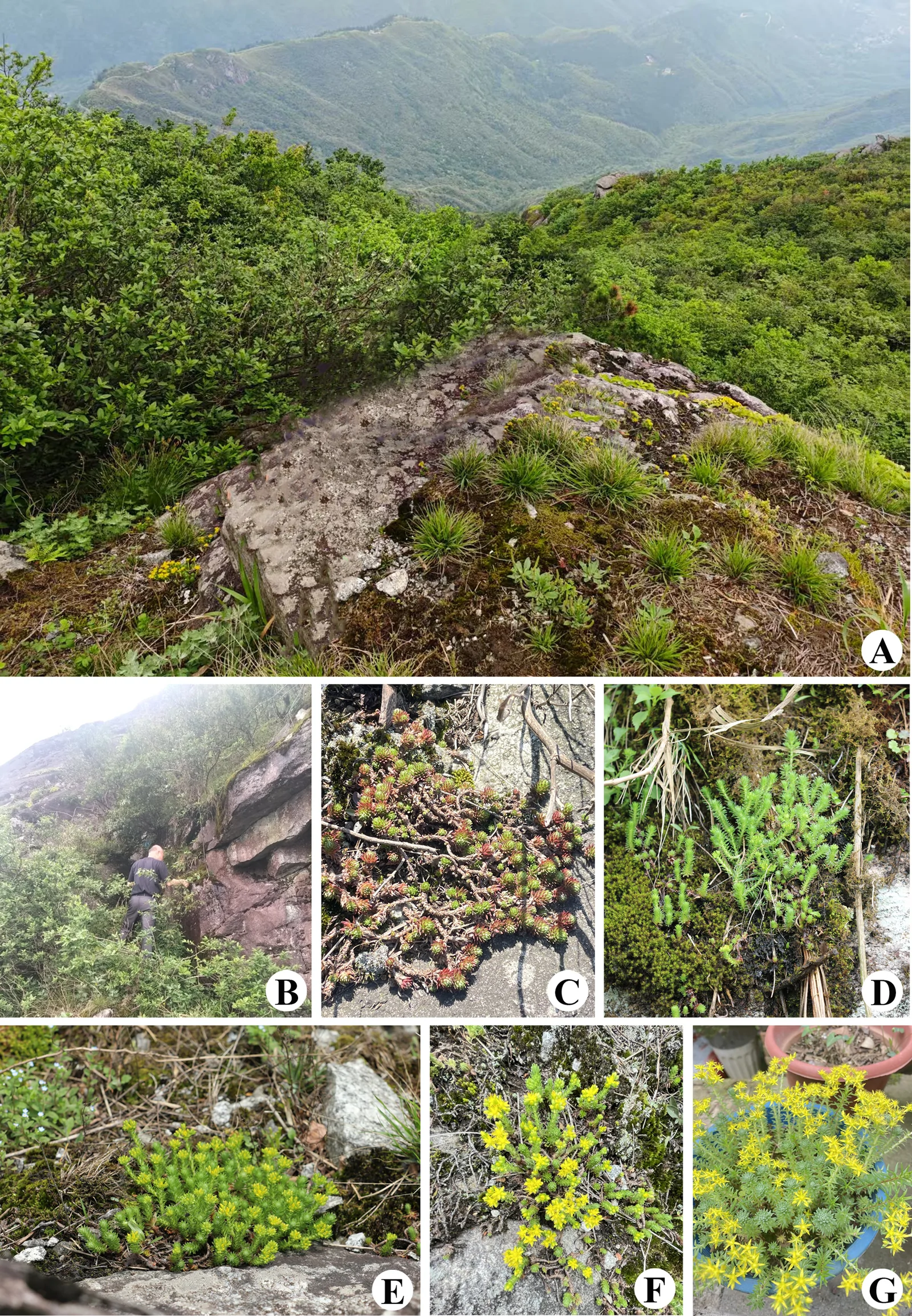
*Sedum hengshanense*. **A, B**. Habitat on the open rocky slope; **C**. Habit in winter; **D**. Habit in spring; **E, F**. Mature plants at flowering stage; **G**. Mature plants under cultivation.

##### Description.

Perennial herbs, entirely glabrous. ***Stems*** all fertile, succulent; older stems prostrate, bearing persistent withered leaves; young stems arising from older stems, 6–12 cm tall. ***Leaves*** alternate, imbricate, oblong-lanceolate, not twisted when young, 7–10 mm long, 1.5–2.0 mm wide, ca. 0.6 mm thick; apex acute; basal leaf spur acuminate, 1.0–1.4 mm long; margins papillate, especially conspicuous in the upper part; succulent. ***Inflorescence*** cymose, terminal, 3–5-branched, 2–4 cm in diam.; bracts leaf-like, similar to the leaves. ***Flowers*** numerous, sessile, actinomorphic, 0.9–1.2 cm long. ***Sepals*** 5, unequal, oblong-lanceolate, 2.5–5.0 mm long, 1.0–1.2 mm wide; apex shortly acuminate, conspicuous papillae; base spurred, spur ca. 0.8 mm long; green, fleshy. ***Petals*** 5, elliptic-lanceolate, 5–6 mm long, 1.2–1.5 mm wide; apex acuminate, shortly mucronate at the tip; basally connate for ca. 0.3 mm; yellow, fleshy. ***Stamens*** 10; antepetalous stamens 3.5–4.2 mm long, antesepalous stamens 3.9–4.4 mm long. ***Pollen*** tricolpate, ellipsoid, 26–28 μm long, 12–13 μm wide, exine with irregularly etched ornamentation. ***Carpels*** 5, erect, adaxially gibbous, lanceolate, basally connate for ca. 0.5 mm; ca. 4 mm long, ca. 0.9 mm wide; yellow. ***Nectar scales*** 5, broadly spatulate to subtrapezoid, apex obtuse, ca. 0.5 mm long, ca. 0.3 mm wide, white. ***Follicles*** obliquely divergent, gibbous, many-seeded. ***Seeds*** ellipsoid, surface densely papillate, 0.6–0.8 mm long, 0.2 mm wide.

**Figure 3.**
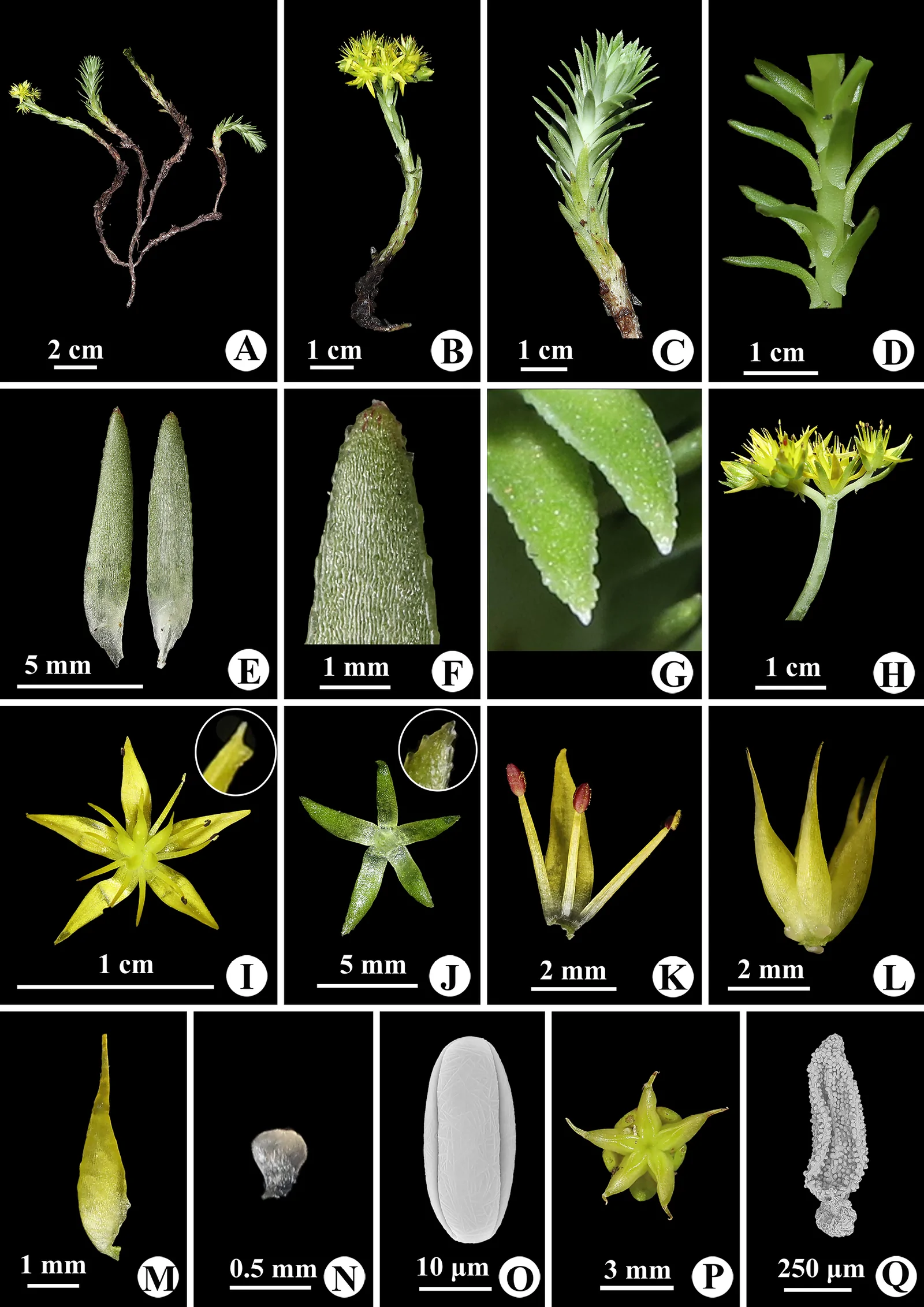
*Sedum hengshanense*. **A, B**. Habit; **C**. Fertile stem; **D**. Stem and leaves; **E**. Leaves; **F, G**. Upper portion of the leaf blade (showing marginal papillae); **H**. Inflorescence; **I**. Flower (sepals removed; inset showing the mucronate apex of petal); **J**. Sepals (inset showing papillate sepal apices); **K**. A petal and the stamens; **L**. Pistil and nectar scales; **M**. Carpel; **N**. Nectar scale; **O**. Pollen; **P**. Fruit; **Q**. Seed (not well developed).

##### Phenology.

Flowering from April to May. Fruiting from May to June.

##### Distribution and habitat.

**Figure 4.**
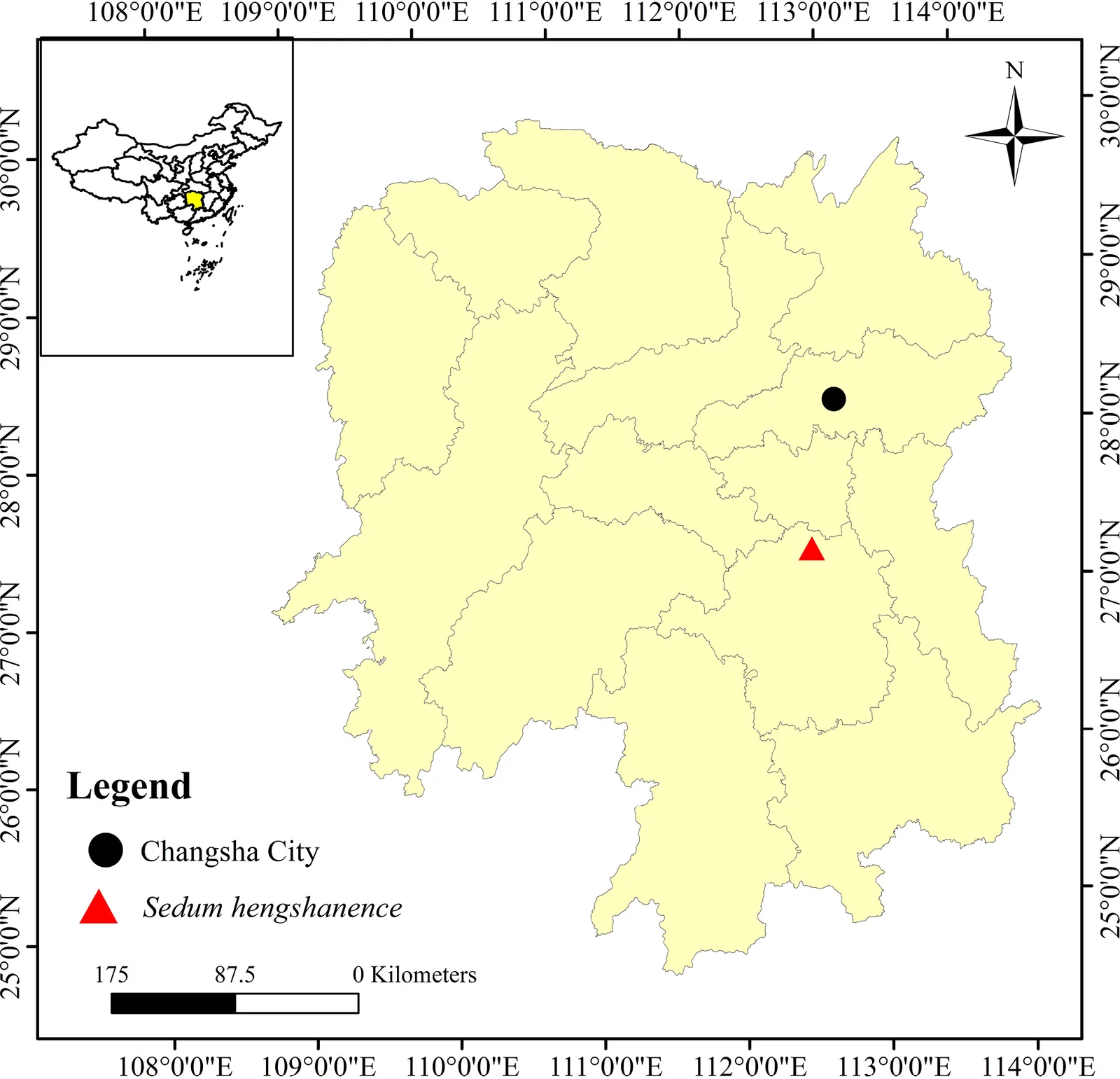
Distribution of *Sedum hengshanense* in Nanyue District, Hunan Province, China.

*Sedum hengshanense* is distributed in Hengshan Mountain, central Hunan Province, China (Fig. 4), at elevations of 480–1250 m. It grows mainly on moist rocky cliffs, especially above 1000 m.

##### Additional specimens examined

**(*paratypes*)**. • Hunan: Nanyue District, Hengshan Mountain, Zhurong Peak, 27.293782°N, 112.694610°E, alt. 1230 m, on rocky slopes, 27 Apr 2025, *J.L.Xia QS16* (HIB!); • the same locality, Hongqidianzhan, 27.315003°N, 112.672141°E, alt. 481 m, on rocky slopes, 6 May 2026, *J.L.Xia QHN01* (HIB!); • the same locality, Huixianqiao, 27.29469°N, 112.69115°E, alt. 1200 m, on rocky slopes, 9 Jun 2026, *J.L.Xia QHN02* (HIB!); • the same locality, Nantianmen, alt. 1100 m, on rocky slopes, 9 Jun 1964, *C.M.Chen ex J.S.Yue 1595* (NAS!).

##### Conservation status.

Three populations were documented in the wild, comprising fewer than 1,000 mature individuals. Two of these, located near the summit of Zhurong Peak, are vulnerable to anthropogenic disturbance associated with tourism development. Following the IUCN Red List Categories and Criteria (IUCN 2024), this new species is here assessed as Vulnerable (VU) under criteria C2a(i) and D1.

##### Etymology.

The specific epithet ‘*hengshanense*’ refers to Hengshan Mountain (Hunan), the type locality where the species was discovered.

## Supplementary Material

XML Treatment for Sedum hengshanense

## Additional information

### Artificial Intelligence (AI) use

The authors accept full responsibility for the content of the manuscript, including the disclosure of any use of AI.

No AI tools were used in the preparation of this manuscript.

### Author ORCIDs

X.-D. Qiu https://orcid.org/0009-0007-2464-6921

F.-D. Zhang https://orcid.org/0009-0004-8943-8419

S.-W. Wang https://orcid.org/0000-0002-8785-5065

N. Wei https://orcid.org/0000-0002-0313-3912

Q.-F. Wang https://orcid.org/0000-0001-9143-8849

### Data availability

All of the data that support the findings of this study are available in the main text.
